# Liquidity Spillover in International Stock Markets through Distinct Time Scales

**DOI:** 10.1371/journal.pone.0086134

**Published:** 2014-01-20

**Authors:** Marcelo Brutti Righi, Kelmara Mendes Vieira

**Affiliations:** Federal University of Santa Maria, Department of Business, Santa Maria, Rio Grande do Sul, Brazil; University of Warwick, United Kingdom

## Abstract

This paper identifies liquidity spillovers through different time scales based on a wavelet multiscaling method. We decompose daily data from U.S., British, Brazilian and Hong Kong stock markets indices in order to calculate the scale correlation between their illiquidities. The sample is divided in order to consider non-crisis, sub-prime crisis and Eurozone crisis. We find that there are changes in correlations of distinct scales and different periods. Association in finest scales is smaller than in coarse scales. There is a rise on associations in periods of crisis. In frequencies, there is predominance for significant distinctions involving the coarsest scale, while for crises periods there is predominance for distinctions on the finest scale.

## Introduction

The central feature of the operation of capital markets is the ability to buy or sell large quantities of assets fast and at a low cost. This feature is known as the market liquidity. In recent years, it has been witnessed a surge of interest in financial market liquidity and its relation to asset prices. Since the seminal work of [1] empirical literature has recognized that asset illiquidity has significant impact on security pricing. In that sense, some works have documented the role of liquidity as a determinant of expected returns [2]–[8], while others relate liquidity risk, which is basically the possibility of loss of an asset value due to low liquidity [9]–[11].

Once stock returns and the cost of capital of firms are influenced by liquidity, understanding its variation is of fundamental relevance. While early studies focused principally on cross-section [12]–[16], recent works have shifted their focus towards studying time series properties of liquidity [17]. Studies on this subject focused mainly on co-movements in trading activity and liquidity [18]–[26] and commonalities in daily aggregate spreads and depths in equity and Treasury bond markets over an extended period [27]–[29].

Such co-movements and commonalities are known in literature as liquidity spillovers. Understanding the reason for such liquidity spillovers is of broad interest because it can be clarifying on sudden and short systematic liquidity crises. Nonetheless, the liquidity spillover causes are not yet well understood. Some explanations for this question are systematic variations in demand or supply for liquidity. In that sense, some studies attest that financial constraints constitute a systematic liquidity factor because they affect liquidity providers in different securities at the same time [30]. Another related explanation is that a decline in the capital available to financial intermediaries active in multiple securities can trigger an increase in risk aversion, impairing the supply of liquidity in these securities [31].

Despite this literature, there is a lack of studies verifying liquidity spillovers in distinct scales. Since it is likely that there are different decision-making time scales among traders, the true dynamic structure of such spillovers will vary over different time scales associated with those different horizons. Corroborating with this argument, there is empirical evidence that the liquidity premium varies in different time scales (see for example, [32]). Thus, assessing this question can elucidate patterns on liquidity co-movements that previous investigations, which focused only on the short run and the long run, could not.

Given the ability to partition each variable into components of different scales, it is possible to provide a simple and intuitive way to distinguish between investor’s profiles, such as speculators (very short time, represented by finest scales in high frequency) and fundamentalists (long term periods, represented by coarse scales in low frequency). This conception was validated in many studies, as the most recent performed by [33]–[36].

In that sense, the current paper has as main objective to identify liquidity spillovers through different time scales. The current approach is based on a wavelet multiscaling method that decomposes a given time series on a scale-by-scale basis. This study is more related with those that assess co-movements in stock market liquidity. To that, we decompose daily data of returns and volume from U.S., British, Brazilian and Hong Kong stock markets indices in order to calculate the scale correlation between their illiquidities, considering crisis and non-crisis periods. The sample corresponds to the period from June 2005 to September 2012, and it is divided in order to consider non-crisis and crisis periods.

The main contributions of the current paper to academic and practical fields are: i) the identification of liquidity spillovers in different scales, making it possible to realize if there are distinct co-movements patterns for time scales which corroborate or do not corroborate with previous literature results; and ii) the extension of wavelet analysis for financial econometric field, once this technique, largely defunded in economics and finance, still was not applied for this purpose in financial literature.

The remainder of this paper is structured as follows: section 2 brings a literature review on liquidity spillovers; section 3 presents definitions of wavelet multiscaling analysis, as well as some financial literature; section 4 exposes data and empirical procedures utilized in this paper; section 5 presents the obtained results and its discussion; section 6 concludes the paper.

### Liquidity Spillover

Since systemic lack of liquidity is a strong vestige of financial crisis or even credit collapses, to understand the multivariate dynamics between financial assets and markets liquidity has attracted much attention over the years. Theoretical economic explanations for these systematic variations rely on the demand [30] or supply [31] for liquidity. Regarding to empirical evidence, studies firstly focused mainly on cross-section analysis of liquidity spillovers, especially because of the limitations of the available data and computation techniques [12]–[16].

Despite the simplicity of cross-sectional data, the most recent and relevant work on liquidity spillover has been dominated by time series analysis. This kind of study can be divided into two main economic motivations. The first focuses on co-movements in trading activity and liquidity on stock markets, while the second, on commonalities in daily aggregate spreads and depths in equity and Treasury bond markets over an extended period. As highlighted by [17], such co-movements and commonalities are known in academic and practical fields as liquidity spillovers. The current paper is more related with the first class of studies, regarding the temporal analysis of liquidity patterns. In the following, we present, with some degree of details, the related literature of these two groups of studies on liquidity temporal dynamics. Regarding to co-movements, due to the wide existing literature, for parsimony, we present here some classical papers that began studies on this topic, followed by some of the most relevant researches performed on recent years.

[18] is the seminal work regarding co-movements in liquidity. They indicate that quoted spreads, quoted depth, and effective spreads co-move with market and industry-wide liquidity. Their results remain even after controlling individual liquidity determinants, such as volatility, volume, and price. This initial work motivated the analysis of common factors in liquidity. In that sense, [21] document the presence of a systematic, time-varying component of liquidity. At the time of their paper, neither the inventory nor the asymmetric information-based approach to liquidity explained this component. Contemporaneously, [20] utilizing data of 30 Dow stocks found through principal component analysis and canonical correlation that both returns and order flows are characterized by common factors. This common variation is present in various liquidity proxies and market depth coefficients. Motivated by these co-movements between liquidity and stock returns, [19] analyze the relationship between expected equity returns and the level as well as the volatility of trading activity, expecting a positive influence which represents a premium for liquidity risk. However, authors document a negative and strong cross-sectional relationship between stock returns and the variability of dollar trading volume and share turnover.

With respect to more recent developments on this field, [22] latent factor models of liquidity, aggregated across various liquidity measures. Based on the idea that systematic liquidity is a pricing factor, it emerges the possibility of prediction of prices, which is linked to market efficiency, as [24] analyze. [26] examine the relative importance of traders in driving commonality inflows of orders, returns, and liquidity. The other line of studies on spillover effects is related to commonalities in daily aggregate spreads and depths in equity and Treasury bond markets, as pointed before. Once this paper is more related to the other line of studies, we only mention here some of the most relevant papers on this theme, such as [27]–[29], among others.

Regarding to theoretical explanations about the liquidity systematic factors found on literature, some authors made an analysis based on demand side-theory, which suggests that liquidity commonality arises from the behavior of investors and traders. Studies related to the demand side include [25], [37]–[39]. In another side, there is the supply-side theory which suggests that liquidity commonality arises from liquidity providers information sharing and capital constraints. Studies in this perspective include [27], [40]–[41].

Despite this vast literature on economic and financial empirical evidences about liquidity spillovers, there is a lack of studies verifying liquidity spillovers in distinct scales. Since there are different decision-making time scales among traders, the true structure of such spillovers will vary over different time scales associated with those different horizons. In rare collaboration to this subject, [23] examine intraday returns and liquidity patterns of Japanese exchange-traded funds. Their findings suggest that some commonality exists in the returns and liquidity of these apparently different assets. More specifically, they find some distinct patterns in distinct data scales (intraday, daily and monthly) in the sense that there was evidence of intraday spillover in the mean, volatility and depth, but daily spillover is not observed. However, the approach by [23] is not able to isolate each frequency of other scale effects. The current approach used in this paper is based on a wavelet multiscaling method that decomposes a given time series on a scale-by-scale basis, which is able to isolate properly these effects. More details on wavelet techniques are given on section 3, while the empirical procedures are explained on section 4.

### Wavelets

Wavelets, as suggested by its denomination, are small waves. This term was created in the geophysics literature by [42]. However, the evolution of wavelets occurred over a significant time scale and in many disciplines, and their background can be found in [43], [44], among others. Given its ability to partition each variable into components of different scales, it can provide a simple and intuitive way to distinguish multiscale interdependence.

Basic wavelets are characterized into father and mother wavelets, which are represented by formulations (1) and (2), respectively.
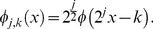
(1)

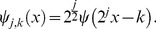
(2)


Where 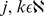, 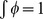, 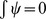 and 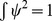. For any function *f* that belongs to a class of functions that are square-integrable, we may write uniquely:
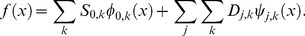
(3)


In (3), 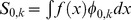 and 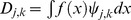 are the Smooth and Detail component wavelet coefficients. Thus, consider a time series *f*(*t*), which we want to decompose into various wavelet scales. Given the father wavelet, so that its dilates and translates constitute orthonormal bases for all the subspaces that are scaled versions of the initial subspace, it is possible to form a Multiresolution Analysis (MRA) for *f*(*t)*. The wavelet function in (3) depends on two parameters, scale and time: the scale or dilation factor *j* controls the length of the wavelet, while the translation or location parameter *k* refers to the location and indicates the non-zero portion of each wavelet basis vector.

The usual approach for this multiresolution analysis is the Discrete Wavelet Transform (DWT). DWT is restricted to sample sizes to a power of 2, i.e., for *j* levels it is necessary a sample of size *2^j^*. In order to overcome this and other difficulties associated with the DWT, in this study we adopt the maximum overlap discrete wavelet transform (MODWT), a highly redundant linear filter that transforms a series into coefficients related to variations over a set of scales [43]–[45]. The MODWT allows alignment of wavelet scaling and detail coefficients with the original time series, and it can also handle any sample size.

Differently from DWT, MODWT is nonorthogonal and has a high level of redundancy, retaining down sampled values at each level of the decomposition that would be discarded by the DWT. The level *j* coefficients have a width 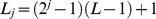, where *L* is the width of the *j = *1base filter. In other words, the MODWT treats time-series as if they were periodic, using “circular boundary conditions.” There are 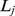 wavelet and scaling coefficients that are influenced by the extension, which are referred to as the boundary coefficients.

Based on wavelet coefficients 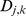, it is possible to estimate univariate and multivariate moments for each scale. Below, we define, based on [45], the wavelet variance, skewness, kurtosis, covariance and correlation. The wavelet variance at scale *j* is defined as the expected value of 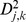 if we consider only the nonboundary coefficients. An unbiased estimator of the wavelet variance for function *f*(*x*) at scale *j* is formed by removing all coefficients that are affected by boundary conditions. The wavelet variance decomposes the variance of a process on a scale-by-scale basis (at increasingly higher resolutions of the signal) and it allows exploring how a signal behaves at different time-horizons. Similarly, the wavelet skewness and kurtosis can be defined on a scale-by scale basis. Also, extending to the bivariate case, covariance and correlations can be calculated. Further information about the calculation of wavelet moments can be found in [33], for instance.

In empirical literature on economics and finance, wavelet analysis has been previously applied to the examination of foreign exchange rates [46]–[47], decomposition of economic relationships of expenditure and income [48], systematic risk in a capital asset pricing model [49], identification of financial contagion [35], [50], [51], de-noising option prices [36], hedging techniques with futures [33], [52];, identifying volatility spillovers [34].

## Methods

For a detailed empirical illustration, we use daily data of closing prices and dollarized trading volumes from U.S. (S&P500), British (FTSE100), Brazilian (Ibovespa) and Hong Kong (HSI) stock market indices from June 2005, to September 2012, totalizing 1820 observations. The first two markets are developed countries, initially affected by the two crises present in sample period (sub-prime and Eurozone). The last two markets are emerging and potential contagion targets, once they are used for international diversification. In this study, we prefer to use only one market of each continent in order to avoid too many dependence due to geographic factors. These indices were chosen because they represent the stock market activity of these countries. Regarding to periodicity, daily data provide more observations, which is a fundamental feature in the analysis of distinct time scales.

For the sample division of crisis and non-crisis periods, we proceed as follows: For sub-prime crisis period we consider trading days starting in August 1, 2007, which is the real state bubble “burst”, until July 13, 2010, which is the beginning of the Eurozone debt crisis, as pointed by [53] based on structural change tests. Thus, the Eurozone crisis is represented by trading days from June 14, 2010 to September 28, 2012. Trading days before the sub-prime crisis until June 1, 2005 are considered non-crisis period.

The liquidity measure adopted in this study is adapted from that proposed by [5]. This measure, represented by *I_i,t_* = *|r_i,t_|*/*V_i,t_*, actually identifies the *Illiquidity* of a determined asset. *I_i,t_* is the illiquidity of a market *i* in period *t*; *|r_i,t_|* represents market *i* absolutelog-return in period *t*; *V_i,t_* is the financial volume of the market *i* in period *t*. Daily volumes are standardized to basis 100, in order to be in the same magnitude for all markets. We also perform the same analysis without this standardization, reaching the same results. In sum, this measure represents the market movements (returns) adjusted by the activity level (volume). The financial logic of this analysis is that during periods of low liquidity, return ranges (volatility) are more intense.

We perform the MODWT in all illiquidity series. Thus, time-scale decomposition analysis is conducted for non-crisis, sub-prime crisis and Eurozone debt crisis, through MODWT, as explained in section 3. Previous studies ([54], for example) on high frequency data have shown that a moderate-length filter such as L = 8 is adequate. Thus, we use the Daubechies compactly supported least asymmetric wavelet filter of length L = 8 (LA8), based on eight non-zero coefficients with reflecting boundary conditions [55]. We also use for comparison purposes, the Daubechies extremal phase wavelet filter of distinct lengths, for a matter of robustness. The same choice is applied, for instance, in [35]. As the sample splits of this research have around 2 or 3 years of data, it was possible to decompose it in 9 scales (2^9^ = 512 observations, so for 10 scales it would be necessary at least 2^10^ = 1024 observations). However, in order to take market pace into account, we restricted the analysis for the first 6 levels or 1 to 32 trading days, with level *n* referring to 2*^n-1^* trading days.

As pointed out before, the wavelet coefficients can be straightforwardly manipulated to obtain recognizable statistical quantities such as wavelet variance, wavelet covariance, and wavelet correlation. In that sense, we compute wavelet correlation for all pairs of markets in all scales. Once these correlation estimates have standard errors, it is possible to compute confidence intervals for them, considering a desired significance level. In this work we use the usual 5% level. Thus, we conclude that there is a change of liquidity spillover if the intersection between wavelet correlations confidence intervals is null.

For example, if the wavelet correlation between the illiquidity measures of a pair of markets in scales 5 is out of the confidence interval for the same correlation in scale 6, we infer that correlations in scale 5 and 6 are significantly different at 5% level. Note that this kind of hypothesis testing can be conducted for discrepancies at the same scale for the distinct split periods of the sample. For example, the correlation coefficient at scale 5 during non-crisis period can be significantly different from scale 5 correlation coefficient in a crisis period. Thus, we perform tests in order to see if there are differences on wavelet correlations of markets illiquidity in distinct scales inside each split period and in the same scale for different split periods.

## Results and Discussion

**Figure 1:**
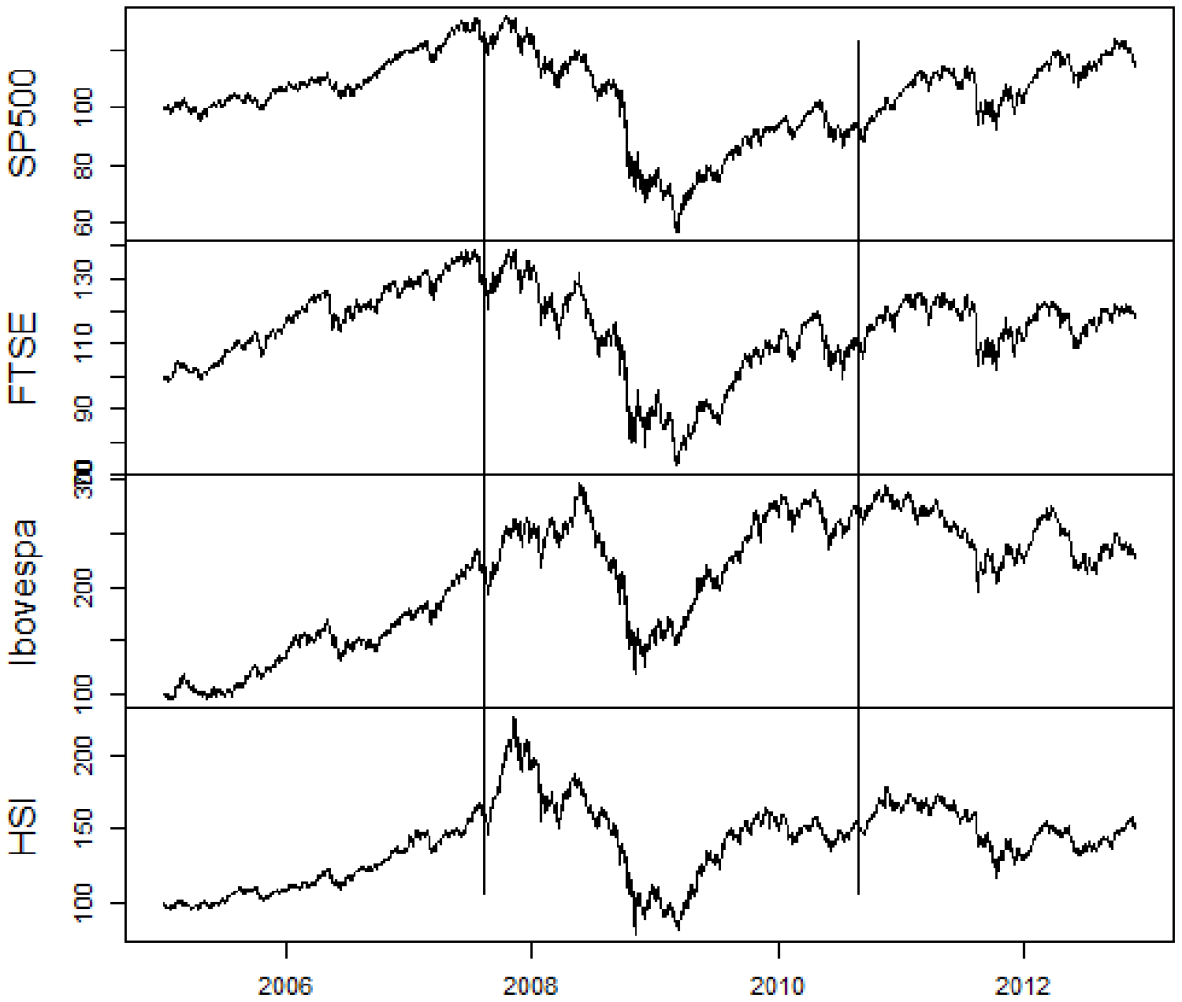
Daily prices on basis 100 of S&P500, FTSE100, Ibovespa and HSI from January 2005 to November 2012. Vertical lines represent the subdivision into non-crisis, Sub-prime crisis and Eurozone crisis.

**Figure 2:**
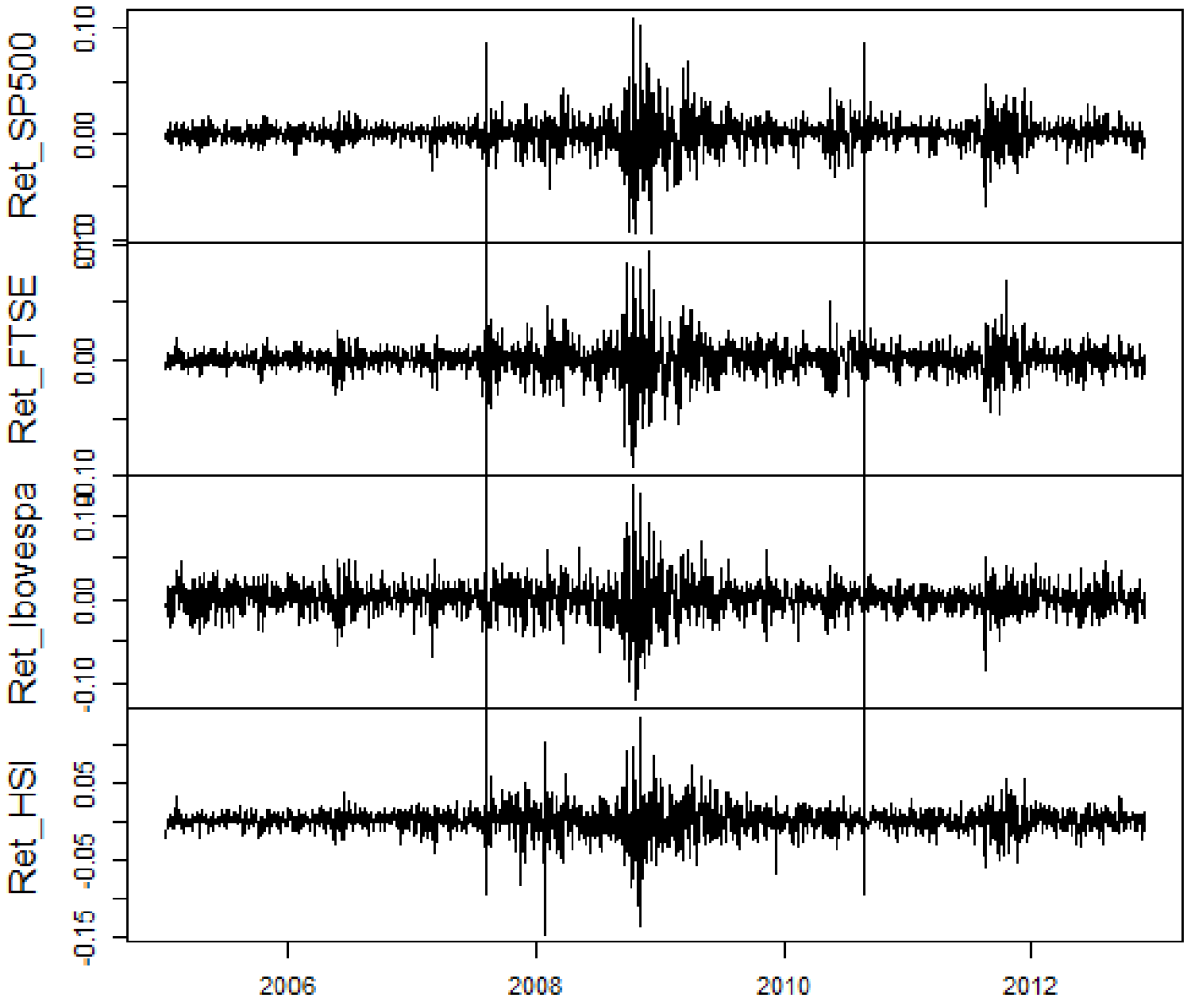
Daily log-returns of S&P500, FTSE100, Ibovespa and HSI from January 2005 to November 2012. Vertical lines represent the subdivision into non-crisis, Sub-prime crisis and Eurozone crisis.

**Figure 3:**
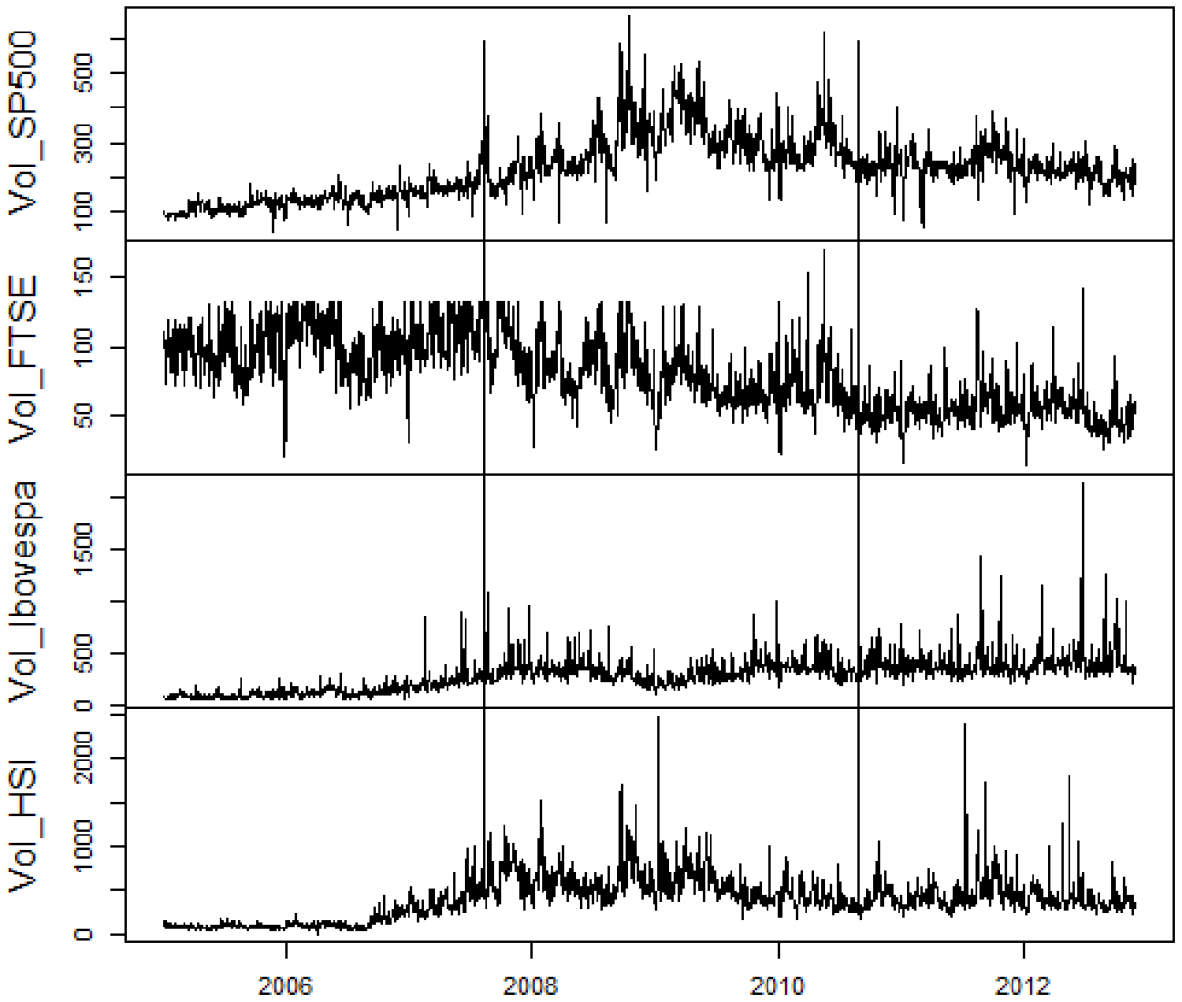
Daily volumes on basis 100 of S&P500, FTSE100, Ibovespa and HSI from January 2005 to November 2012. Vertical lines represent the subdivision into non-crisis, Sub-prime crisis and Eurozone crisis.

**Figure 4:**
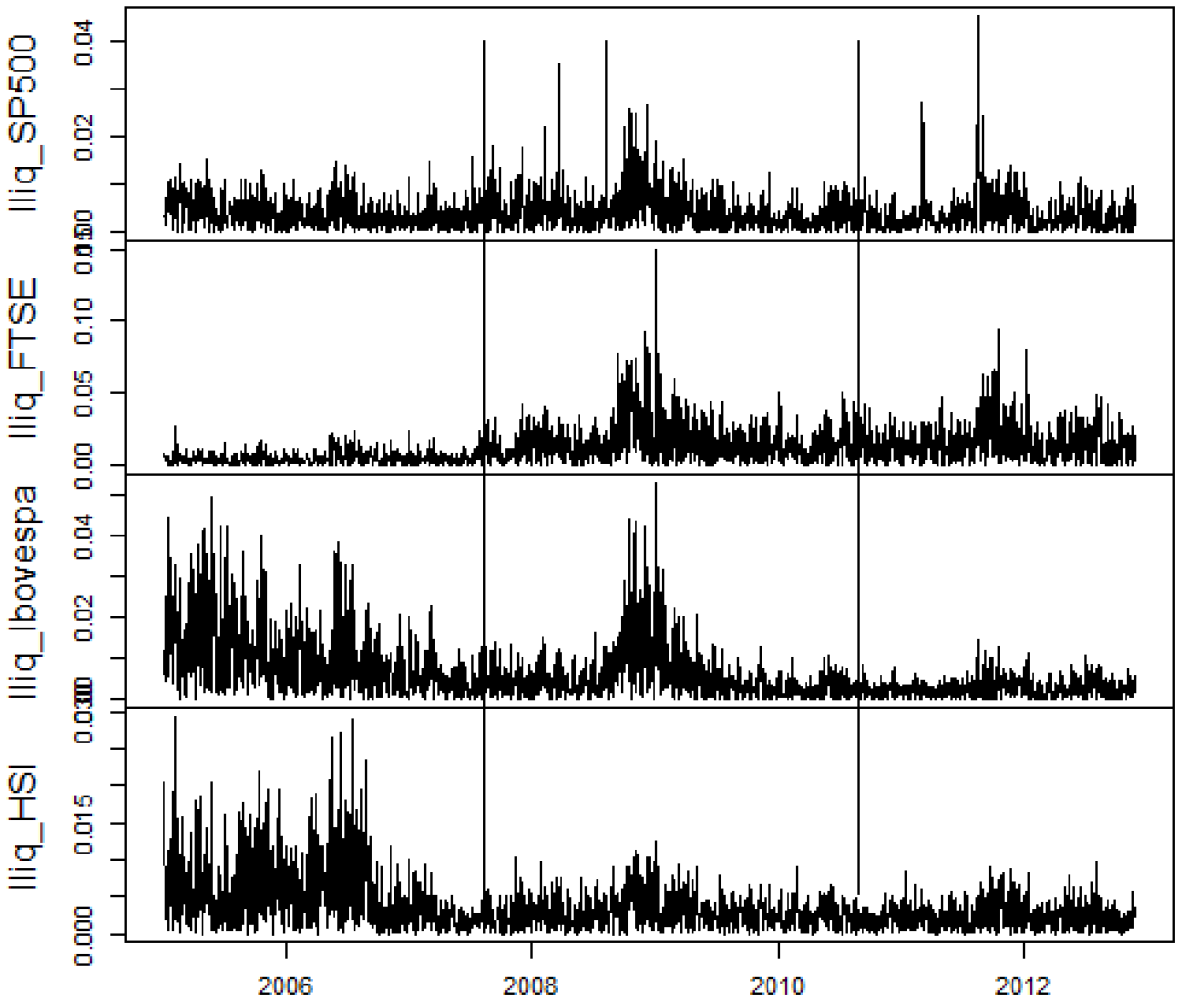
Daily illiquiditiess of S&P500, FTSE100, Ibovespa and HSI from January 2005 to November 2012. Vertical lines represent the subdivision into non-crisis, Sub-prime crisis and Eurozone crisis.

At first, we present the utilized data. For a visual comprehension, Figs. 1, 2, 3, 4 contain, respectively, daily prices, log-returns, volumes and illiquidities of the analyzed markets. The non-crisis period (from the beginning to the first vertical line) corresponds to a rising trend in market prices and low volatile market log-returns, with smooth growth and volumes slightly volatile (except FTSE100). In the sub-prime period (between the first and the second vertical lines), there is a falling trend in market prices and huge log-returns volatility clusters and larger volume levels. The Eurozone crisis (from the second vertical line to the end) presented an initial fall in prices and volatile returns. After a relative recovering and a calm period, there were major falls in prices and volatility clusters. Volume levels were a little lower than during sub-prime crisis period. Figs. 1, 2, 3 elucidate distinct occurred behaviors during the whole sample period.

Regarding to illiquidities, which are the central variables in this study, plots in Figure 4 exhibit an interesting behavior. In non-crisis periods, developed markets were very liquid, while emerging ones had high illiquidity. Nonetheless, during sub-prime crisis period, developed markets, more affected by turbulence, lost liquidity, while the emerging markets, on the other hand, gained liquidity. In this period, illiquidity levels from all markets were very similar. As on the Eurozone crisis, the pattern of evolution was kept, so emerging markets became proportionally more liquid than developed ones.

This result is intimately linked with the fact that emerging markets had gained importance for international diversification, receiving capital originated from developed markets. The study of [38] corroborate with this findings, in the sense that liquidity spillovers are intense during times of high market volatility and in countries with greater presence of international investors. Furthermore, this logic reinforces the choices made in this study, for sample subdivision and selected markets.

**Figure 5:**
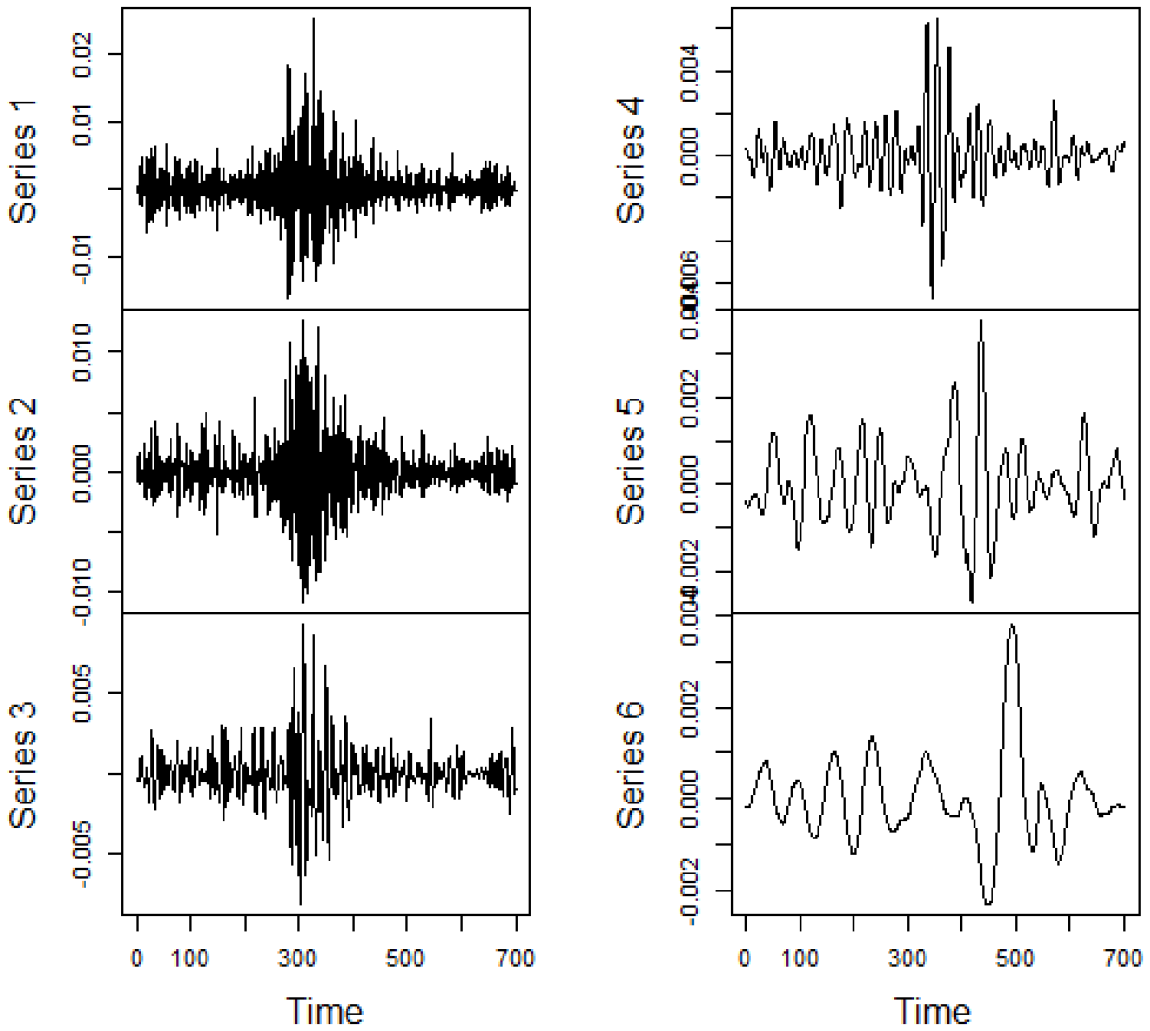
Daily wavelet coefficients of S&P500 during sub-prime crisis period. Series *n* refers to Wavelet level *n* or 2*^n-1^* trading days.

After this initial descriptive analysis, we perform the MODW, as detailed on section 4. Thus, we obtain for each market in each sub-period, 6 series of wavelet coefficients linked with different scales. Under this framework, the result is 4×3×6 = 72 series of coefficients. Due to parsimony, not all series are exhibited. However, for an illustration, Fig. 5 presents the wavelet coefficient series for S&P500 during the sub-prime period. The pattern of decomposition is similar for all the other markets in the three periods. Finest scales bring high frequency information while coarse scales exhibit low frequency information. Scales 1 to 6 represent 1 to 32 trading days. The illiquidity measure used is basically a return adjusted by volume, as explained in section 4. Its behavior is indeed very similar to financial returns, which are understood as stationary time series, at least in the weak form. The volatility clusters are very well-known stylized facts about financial data, and are commonly modeled through GARCH models, for instance. Such clusters are not considered as having a non-stationary behavior by practically every study. In finance, these properties are analyzed and not eliminated. In this study, we made a division by crisis periods, in order to consider this volatility change, which produces very relevant results. Even if this was not the case, wavelet analysis is able to deal with non-stationary data in many cases.

Despite the fact that an individual analysis of wavelet coefficients is not the scope of this paper, it is noteworthy that there are discrepancies between distinct scales and periods. Dispersion is generally greater in finest scales than in coarse ones, reflecting the risk inherent to speculative trading in high frequency. This pattern is similar to that found in [34] when verifying volatility spillovers. Regarding to skewness, there is a predominance of positive values. This result reflects the fact that illiquidity is defined as positive values with an inferior limit but not with a superior one. In relation to kurtosis, some series exhibited leptokurtic behaviors, indicating the occurrence of ‘extreme’ illiquidities.

**Figure 6:**
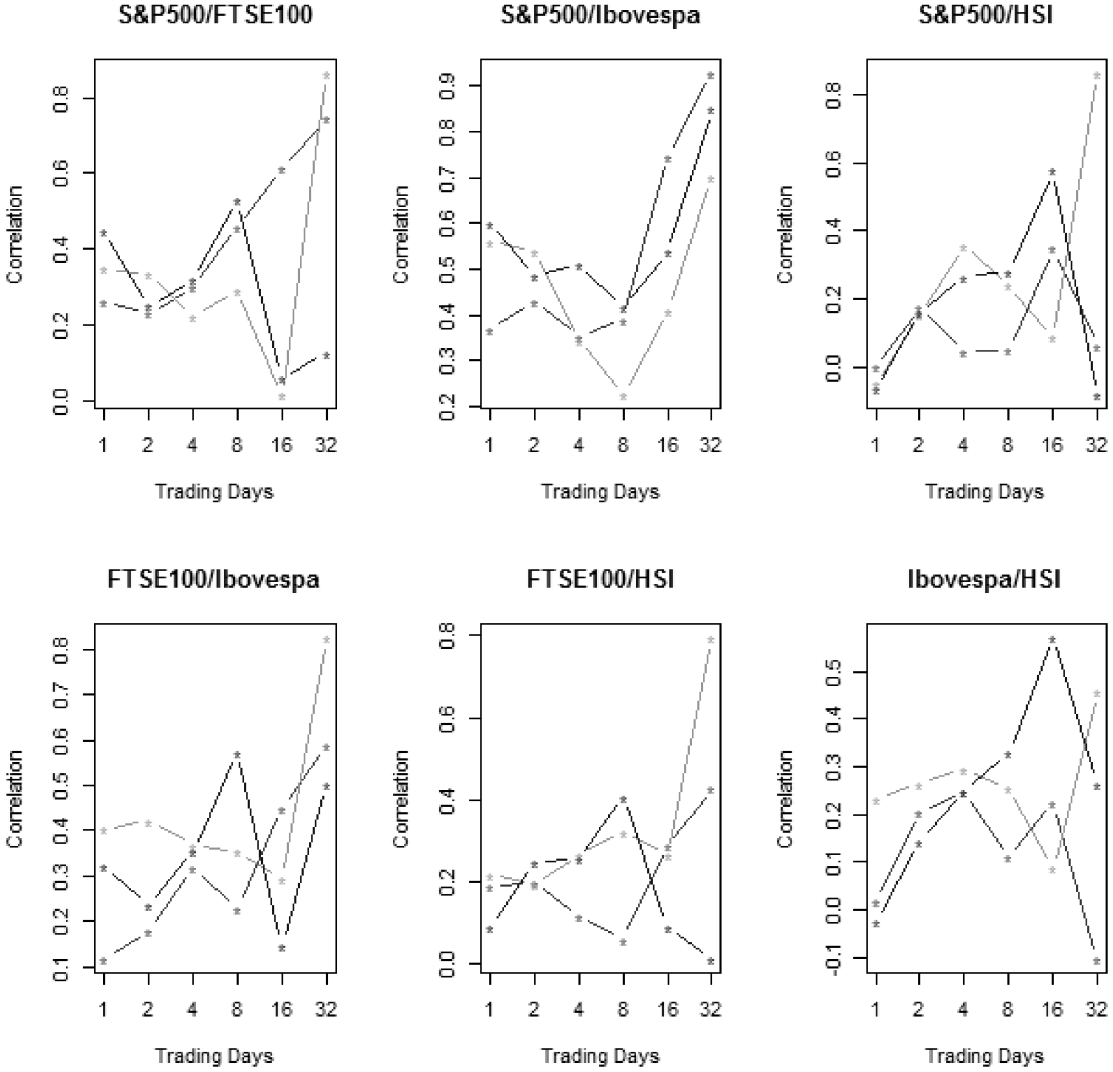
Correlation for S&P500, FTSE100, Ibovespa and HSI wavelet coefficients relationships in non-crisis (middle gray), Sub-prime crisis (weak gray) and Eurozone crisis (strong gray).

After this individual analysis, we turn the focus to the joint analysis of wavelet coefficients. As explained in previous sections, it was calculated, for each pair in each scale, the correlation between coefficients of the illiquidity series for the three periods. Numerical results are presented in Table 1. For a visual comprehension, we plot the same results in Fig. 6.

**Table 1:** Correlations for S&P500, FTSE100, Ibovespa and HSI wavelet coefficients relationships in non-crisis, Sub-prime crisis and Eurozone crisis.

Period	Wavelet Level					
Non-crisis	1	2	3	4	5	6
S&P500|FTSE100	0.2587	0.2301	0.3011	0.4582	0.6134	0.7456
S&P500|Ibovespa	0.3694	0.4297	0.3536	0.3898	0.7469	0.9288
S&P500|HSI	0.0011	0.1748	0.0485	0.0517	0.3492	0.0607
FTSE100|Ibovespa	0.1153	0.1792	0.3165	0.2298	0.4485	0.5868
FTSE100|HSI	0.1907	0.1981	0.1167	0.0594	0.2882	0.4283
Ibovespa|HSI	0.0172	0.2055	0.2478	0.1098	0.2239	−0.1050
**Period**	**Wavelet Level**					
**Sub-prime**	**1**	**2**	**3**	**4**	**5**	**6**
S&P500|FTSE100	0.3474	0.3357	0.2230	0.2898	0.0136	0.8655
S&P500|Ibovespa	0.5611	0.5396	0.3440	0.2246	0.4108	0.7004
S&P500|HSI	−0.0472	0.1565	0.3582	0.2410	0.0889	0.8617
FTSE100|Ibovespa	0.4026	0.4222	0.3670	0.3538	0.2935	0.8260
FTSE100|HSI	0.2183	0.1928	0.2675	0.3320	0.2672	0.7951
Ibovespa|HSI	0.2312	0.2633	0.2946	0.2542	0.0866	0.4565
**Period**	**Wavelet Level**					
**Eurozone**	**1**	**2**	**3**	**4**	**5**	**6**
S&P500|FTSE100	0.4492	0.2489	0.3210	0.5293	0.0592	0.1258
S&P500|Ibovespa	0.6001	0.4843	0.5096	0.4189	0.5370	0.8509
S&P500|HSI	−0.0614	0.1603	0.2667	0.2786	0.5763	−0.0801
FTSE100|Ibovespa	0.3218	0.2359	0.3553	0.5733	0.1462	0.7007
FTSE100|HSI	0.0897	0.2458	0.2557	0.4069	0.0908	0.0107
Ibovespa|HSI	−0.0272	0.1434	0.2478	0.3272	0.5707	0.2634

* Wavelet level *n* corresponding to 2*^n-1^* trading days.

The interested is to analyse two kinds of patterns. Firstly, correlation changes for distinct trading days. Secondly, correlation changes in different periods. In regards to the frequency perspective, there is a pattern, which is common to all bivariate relationships. Association in finest scales is smaller than in coarse scales. This result emphasizes the existence of a long-term strong common factor on the liquidity of international markets. This finding is in consonance with the early work of [21], which documents the presence of a systematic, time-varying component of the liquidity. At the time of their paper, neither the inventory nor the asymmetric information based approach to liquidity explained this component. Perhaps the changes on supply and demand curves inherent to distinct frequency based traders could be an explicative factor so far unexplored for this theme.

Still on the importance of isolating the frequency-based information, results on Table 1 and Fig. 6 indicate that there are associations on markets illiquidity in a daily basis. This result is in contrast with [23] who found evidence of intraday spillovers, but daily spillover was not observed. Again, as for long-term liquidity, considering frequency-based traders on an isolated perspective can lead to distinct information that was not available before.

Differences on illiquidity associations in distinct scales should be linked with their individual dispersions. It is evident that finest scales have larger standard deviations than the coarse ones. This discrepancy on variability is noted by [32], which argue that short-time investors, which correspond to the speculative capital for liquidity, assume more volatile positions than that of the long-term conservator ones. Short time investors seek for a rapid diversification of their portfolios, in addition to the facility to pay off the positions and leave a specific market.

Hence, it is empirically confirmed that there are differences regarding the magnitude of liquidity spillovers in international markets. Thus, this pattern should be considered when one estimates pricing models or even for risk management purposes that incorporate liquidity risk as a prime factor.

At this point, we turn the focus to the effects of periods on liquidity spillovers. Once we have shown the existence of a frequency effect, this period factor is secondary for the purposes of this paper, but it can give an interesting illustration of how financial distresses such as crises can affect liquidity dynamics. Results on Table 1 and Fig. 6 present a tendency of a rise on associations due to crisis periods, in comparison to non-crisis period. With the exception of some relationships in specific scales, the correlation between wavelet coefficients is lower in non-crisis than during Sub-prime and Eurozone crisis. This finding is intimately related to the contagion effect, well defined by [56], which is the elevation on association of markets occasioned by financial turbulence.

Regarding to frequency based analysis contagion is investigated through wavelet techniques by [35], [50]–[51]. These works in general argue that changes in magnitude of markets interdependence is not uniform for distinct time scales. This pattern is corroborated by our findings because there is no homogeneity in changes from non-crisis to crisis through distinct scales.

Afterwards, we show that liquidity spillovers vary both in time scales as in crisis periods. Nonetheless, in order to have a more specific analysis of these findings, we present on Table 2 the most significant correlation differences for the relationships between S&P500, FTSE100, Ibovespa and HSI wavelet coefficients. The presented differences are those that lie on the established criteria of rejection of the null hypothesis of equality of correlation explained in section 4. Firstly, and perhaps the most important, all relationships exhibit some significant difference in liquidity spillovers.

**Table 2:** Most significant correlation differences for the relationships between S&P500, FTSE100, Ibovespa and HSI wavelet coefficients in non-crisis (NC), Sub-prime crisis (SP) and Eurozone crisis (EU).

Relationship	Pairs of wavelet levels with statistically differentvalues for their correlation coefficients, byperiod and pair of financial markets		Wavelet level where the difference of two correlation coefficients is the largest, by pair of periods and pair of financial markets	
S&P500|FTSE100	NC	–	NC| SP	–
	SP	1|6, 2|6, 3|6, 4|6, 5|6	NC| EU	1
	EU	–	SP| EU	–
S&P500|Ibovespa	NC	1|5, 1|6, 2|6, 3|6, 4|6	NC| SP	1
	SP	1|3, 1|4, 2|3, 2|4	NC| EU	1
	EU	1|2	SP| EU	–
S&P500|HSI	NC	–	NC| SP	6
	SP	1|3, 1|6, 2|6, 3|6, 4|6, 5|6	NC| EU	–
	EU	1|2, 1|3, 1|5	SP| EU	–
FTSE100|Ibovespa	NC	–	NC| SP	1
	SP	1|6	NC| EU	1
	EU	2|4	SP| EU	–
FTSE100|HSI	NC	–	NC| SP	–
	SP	1|6, 2|6, 3|6	NC| EU	–
	EU	–	SP| EU	–
Ibovespa|HSI	NC	–	NC| SP	1
	SP	–	NC| EU	–
	EU	1|3, 1|4, 1|5	SP| EU	–

Middle panel: pairs of wavelet levels with statistically different values for their correlation coefficients, by period and pair of financial markets. Right panel: wavelet level where the difference of two correlation coefficients is the largest, by pair of periods and pair of financial markets.

* Wavelet level *n* corresponding to 2*^n-1^* trading days.

In relation to frequency, distinct pairs show differences, but there is predominance for distinctions involving the coarsest scale with 32 trading days. As evidenced in Fig. 6, this is the most discrepant scale. An explanation is the fact that it has the greatest difference in terms of absolute number of days for other scales. Regarding periods, there is predominance for distinctions on the finest scale, corresponding to 1 trading day. This reflects that sensibility of speculators for financial distresses is larger than that for fundamentalist investors. As mentioned before, high frequency traders seek for a rapid diversification of their portfolios, in addition to the facility to pay off the positions and leave a specific market.

## Conclusion

This paper identifies liquidity spillovers through different time scales. The current approach is based on a wavelet multiscaling method that decomposes a given time series on a scale-by-scale basis. We decompose daily data of returns and volume from U.S., British, Brazilian and Hong Kong stock markets indices in order to calculate the scale correlation between their illiquidities, measured as an adaptation of [5]. The sample corresponds to the period from June 2005 to September 2012, and it is divided in order to consider non-crisis, sub-prime crisis and Eurozone crisis.

Descriptive analysis of illiquidities clarify that during non-crisis periods, developed markets were very liquid, while emerging ones had high illiquidity, but this pattern has inverted during the two crisis periods. This result reflects the importance of emerging markets in diversification strategies during these turbulent periods. After decomposition, it was evident that there are discrepancies between distinct scales and periods. Dispersion is generally larger in finest scales. Skewness has predominance of positive values. Kurtosis presents some leptokurtic behaviors, but a predominance of close to zero exceeding kurtosis.

Regarding spillovers, we found that there are correlation changes for distinct scales and different periods. Association in finest scales is smaller than in coarse scales, emphasizing the existence of a long-term strong common factor on the liquidity of international markets. This result extends previous researches, reinforcing the need for isolating scales on liquidity analyses. Regarding to differences in periods, a rise on associations was observed, due to crisis periods, in comparison to non-crisis period. Correlation between wavelet coefficients is lower in non-crisis than during Sub-prime and Eurozone crisis, which is related with contagion.

On a more specific point of view, there is predominance of distinctions involving the coarsest scale with 32 trading days. An explanation for this is the fact that it has the great difference in terms of absolute number of days for other scales. Regarding periods, there is predominance for distinctions on the finest scale, corresponding to 1 trading day. This reflects the sensibility of speculators for financial distresses.

Hence, based on significance tests of wavelet correlations, this study empirically confirms that there are differences regarding the magnitude of liquidity spillovers in international markets. We do not attempt to identify *how* these spillovers occur in the sense that choosing an econometric model could lead to estimation biases. Serial correlations could be considered, for example. This matter is left for future studies. It also stands out for the next researches, the use of other liquidity/illiquidity measures, and other samples. Furthermore, it is possible to verify how the multiscale pattern affects distinct kinds on investors. Finally, one should consider intraday data, for a more detailed behavior of liquidity dynamics in ultra-high scales.
